# Diet-Related Phototoxic Reactions in Psoriatic Patients Undergoing Phototherapy: Results from a Multicenter Prospective Study

**DOI:** 10.3390/nu13092934

**Published:** 2021-08-25

**Authors:** Alessia Pacifico, Rosalynn R. Z. Conic, Antonio Cristaudo, Sergio Garbarino, Marco Ardigò, Aldo Morrone, Paolo Iacovelli, Sara di Gregorio, Paolo Daniele Maria Pigatto, Ayman Grada, Steven Richard Feldman, Egeria Scoditti, Khalaf Kridin, Giovanni Damiani

**Affiliations:** 1 Clinical Dermatology Department, San Gallicano Dermatological Institute, IRCCS, 00144 Rome, Italy; alessia.pacifico@gmail.com (A.P.); antonio.cristaudo@libero.it (A.C.); ardigo.marco@gmail.com (M.A.); aldomorrone54@gmail.com (A.M.); paolo.iacovelli@ifo.gov.it (P.I.); 2 Department of Preventive Medicine, Maryland University, Baltimore, MD 21201, USA; ruzica.conic@gmail.com; 3 Department of Neuroscience, Rehabilitation, Ophthalmology, Genetics and Maternal/Child Sciences, Polyclinic Hospital San Martino Istituto di Ricovero e Cura a Carattere Scientifico (IRCCS), University of Genoa, 16132 Genoa, Italy; sgarbarino.neuro@gmail.com; 4 Clinical Dermatology, IRCCS Istituto Ortopedico Galeazzi, 20161 Milan, Italy; saradigregorio95@gmail.com (S.d.G.); paolo.pigatto@unimi.it (P.D.M.P.); 5 Department of Biomedical, Surgical and Dental Sciences, University of Milan, 20122 Milan, Italy; 6 Department of Dermatology, Boston University School of Medicine, Boston, MA 02118, USA; grada@bu.edu; 7 Wake Forest School of Medicine, Department of Dermatology, Winston-Salem, NC 27157, USA; sfeldman@wakehealth.edu; 8 Institute of Clinical Physiology, National Research Council (CNR), 73100 Lecce, Italy; egeria.scoditti@ifc.cnr.it; 9 Lübeck Institute of Experimental Dermatology, University of Lübeck, 23538 Lübeck, Germany; dr_kridin@hotmail.com; 10 PhD Degree Program in Pharmacological Sciences, Department of Pharmaceutical and Pharmacological Sciences, University of Padua, 35131 Padua, Italy

**Keywords:** psoriasis, diet, vegans, vegetarians, omnivores, phototherapy, NB-UVB, efficacy, precision medicine, exposures, exposome, inflammation

## Abstract

Vegans and vegetarians often consume foods containing photosensitizers capable of triggering phytophotodermatitis. The potential effect of vegan and vegetarian diets on the response of psoriatic patients undergoing phototherapy is not well characterized. We assessed clinical outcomes of vegan, vegetarian and omnivore adult psoriatic patients undergoing band ultraviolet B phototherapy (NB-UVB). In this multicenter prospective observational study, we enrolled 119 adult, psoriatic patients, of whom 40 were omnivores, 41 were vegetarians and 38 were vegans, with phototherapy indication. After determining the minimum erythemal dose (MED), we performed NB-UVB sessions for 8 weeks. The first irradiation dosage was 70.00% of the MED, then increased by 20.00% (no erythema) or by 10.00% (presence of erythema) until a maximum single dose of 3 J/cm^2^ was reached and constantly maintained. All the enrolled patients completed the 8 weeks of therapy. Severe erythema was present in 16 (42.11%) vegans, 7 (17.07%) vegetarians and 4 (10.00%) omnivores (*p* < 0.01). MED was lowest among vegans (21.18 ± 4.85 J/m^2^), followed by vegetarians (28.90 ± 6.66 J/m^2^) and omnivores (33.63 ± 4.53 J/m^2^, *p* < 0.01). Patients with severe erythema were more likely to have a high furocumarin intake (OR 5.67, 95% CI 3.74–8.61, *p* < 0.01). Vegans consumed the highest amount of furocumarin-rich foods. A model examining erythema, adjusted for gender, age, skin type, MED, phototherapy type, number of phototherapies and furocumarin intake, confirmed that vegans had a lower number of treatments. Vegans had more frequent severe erythema from NB-UVB, even after adjustment of the phototherapy protocol for their lower MED. Assessing diet information and adapting the protocol for vegan patients may be prudent.

## 1. Introduction

For more than a decade, psoriasis has been considered as a systemic, inflammatory disease with cutaneous [1,2] and musculoskeletal manifestations [3] enriched by a wide range of comorbidities [4], ranging from respiratory [5,6,7,8] to cardiometabolic ones [9,10].

While obesity and metabolic syndrome are risk factors for psoriasis, the contribution of diet to psoriasis and its treatment outcomes are still not completely defined [11]. Vegans and vegetarians often consume vegetables containing furocumarins (i.e., celery, parsnip, carrot, parsley, citrus and figs), natural photosensitizers and triggers for phytophotodermatitis in both humans and animals [12,13,14,15,16,17,18]. Furocumarins are characterized by a coumarin structure with a furan ring and are traditionally classified into linear (“psoralen type”) and angular (“angelicin type”) types [19]. Furocumarins are well absorbed from food sources and rapidly distributed into several tissues, including skin [19], so phototoxic reactions due to the ingestion of food containing such photosensitizers can occur [20]. 

Narrow-band ultraviolet B phototherapy (NB-UVB) represents a useful pre-biologics therapeutic option for psoriatic patients [21]. NB-UVB phototherapy may induce psoriasis clearance through pleiotropic effects on human cutaneous immunity, including the activation of apoptosis, DNA damage response and repair pathways, cell cycle control/differentiation and inflammation regulation [22]. Circadian rhythmicity disruption is also involved in psoriasis pathophysiology [23] and may influence NB-UVB response [22,24,25]. Furthermore, the concept that diet influences both inflammation and peripheral clocks is well established [16,17,26,27], but the impact of vegan and vegetarian diets on phototherapy remains neglected. Due to the increasing number of vegetarians and vegans in the general population, we explored the effect of these diets in psoriatic patients undergoing NB-UVB.

## 2. Materials and Methods

### 2.1. Study Design

This was a multicenter prospective observational study involving two primary referral phototherapy centers (IRCCS San Gallicano Hospital-Rome, and IRCCS Istituto Ortopedico Galeazzi-Milan, Italy). Patients fulfilling inclusion criteria were enrolled, evaluated (T0) and followed up for 8 weeks (T1). Vegans, vegetarians and omnivores were matched for age, gender, skin phenotype and Psoriasis Area Severity Index (PASI). Saint Rafael Hospital (OSR) local ethical committee approved in 28 May 2021 the study protocol 176/int/2020 and the current study represents a post-hoc analysis. Each patient signed an written informed consent.

### 2.2. Inclusion/Exclusion Criteria

We enrolled adult patients (≥18 years) with plaque psoriasis and Fitzpatrick skin type II-IV with an indication for NB-UVB phototherapy. Psoriatic erythroderma was excluded because it is an absolute contraindication to phototherapy. Patients with psoriatic arthritis that refuse systemic treatments but underwent NB-UVB were included. 

Conversely, patients were excluded in case of (a) previous history of skin cancer or chemotherapy (<5 years before); (b) ongoing therapies potentially aggravating psoriasis or recognized as photosensitizing; (c) undergoing topical anti-psoriatic treatments in the previous 2 weeks or systemic ones in the previous 4 weeks; or (d) acute or chronic infectious comorbidities. 

### 2.3. MED Evaluation and Erythema Quantification

MED testing was performed on normal dorsal skin of each enrolled patient before starting phototherapy using a Multiport UV Solar Simulator 601 (Solar Light CO.INC: Philadelphia, PA, USA) [28]. MED testing was repeated after 8 weeks of phototherapy treatment to evaluate photoadaptation. Before and after the phototherapy course, each patient was evaluated for the erythema index with a skin reflectance measuring instrument (Mexameter MX16, Courage & Khazaka Electronics, Cologne, Germany).

MED was calculated more than 6 hours after the last meal to prevent furocumarins absorption peak [19].

### 2.4. Phototherapy Protocol

NB-UVB was delivered by a PUVA Combi Light PCL 8000 phototherapy booth (Heverlee, Belgium) equipped with 48 Phillips^®^ TL100 W/01 tubes in both centers, and both used the same dosimeter. The fluence in the center was 15 mW/cm^2^, as measured with a Waldmann Variocontrol dosimeter (WaldmannMedizintechnik GmbH, Villingen-Schwenningen, Germany). The initial irradiation dose was chosen based on a subject’s minimal erythema dose (MED). 

The first irradiation dosage was 70.00% of the MED, which was then increased by 20.00% (in the absence of erythema) or by 10% (in case of a minimal perceptible onset erythema) until a maximum single dose of 3.00 J/cm^2^ was reached, after which the dose was constantly maintained. Irradiations were administered three times weekly for up to 8 weeks. In the case of patients susceptible to develop a severe erythema, dosing was reduced to two treatments per week.

### 2.5. Dietary Evaluation

During the dermatological visit, dietary information was recorded and patients were classified as vegans, vegetarians or omnivores using the following definitions:

▪ Vegans where patients “ate only all kinds of fruits, vegetables, nuts, grains, seeds, beans and pulses” [29];
▪ Vegetarians or “fully vegetarians” where patients “never ate meat, poultry and fish, or ate these foods less than once a month” [30];
▪ Omnivores if they patients not represented by the previous classifications.

We asked patients to list and weigh each food they consumed in a diet diary for a week. From this information, we identified the approximative quantities of polyphenols [31], carotenoids [32], astaxanthins [33] and furocumarins [34] consumed using converting tables already present in the literature. Then, we divided the obtained quantitatives for each evaluated substance into tertiles and named the first tertile as “low” (“none” in case of no intake), the second one as “intermediate” and the third one as “high” consumption. 

### 2.6. Statistical Analysis

Demographic and food intake characteristics between omnivores, vegetarians and vegans were compared using the t-test for continuous variables and the chi-square test for categorical variables. Association between erythema and diet was further examined using a linear regression adjusting for gender, age, skin type, MED, phototherapy type, number of therapies and furocumarin use. 

## 3. Results

### 3.1. Clinical Data and Demographics

We enrolled 119 psoriatic patients (40 omnivores, 41 vegetarians and 38 vegans) and all of them completed the 8 weeks of therapy. There were no differences in gender (*p* = 0.68), age (*p* = 0.79), skin type (*p* = 0.43), number of phototherapy treatments (0.40) or baseline (T0) PASI (*p* = 0.55). 

Remarkably, after 8 weeks of NB-UVB, there was a statistically significant difference in PASI between omnivores, vegans and vegetarians (1.95 vs. 8.87 vs. 4.59, *p* < 0.01) (Table 1).

### 3.2. Erythema and MED

Severe erythema was present in 16 (42.11%) vegans, 7 (17.07%) vegetarians and 4 (10.00%) omnivores (*p* < 0.01). In total, 19 (47.50%) omnivores and 14 (34.15%) vegetarians experienced no erythema, whilst all vegans experienced some degree of erythema. Patients with severe erythema underwent fewer phototherapy sessions. MED was lowest among vegans (21.18 ± 4.85 J/m^2^), followed by vegetarians (28.90 ± 6.66 J/m^2^) and omnivores (33.63 ± 4.53 J/m^2^, *p* < 0.01). 

### 3.3. Diet Photoactives and Their Impact

Interestingly, 21 (55.26%) vegans consumed high doses of furocumarins, followed by 4 (10.00%) vegetarians and no omnivores (*p* < 0.01). Astaxanthines consumption was high in about 23 (29.11%) vegans and vegetarians, but only in 1 omnivore (*p* < 0.01). High polyphenol consumption was reported by 14 (36.84%) vegans, 11 (26.83%) of vegetarians and only 1 omnivore (*p* < 0.01). There was no difference in carotenoid intake between vegetarians, vegans and omnivores (*p* = 0.31) (Table 2). Only two omnivores consumed a high number of vegetables.

A model examining erythema, adjusted for gender, age, skin type, MED, phototherapy type, number of phototherapies and furocumarins intake, confirmed that vegans had a lower number of treatments. Patients with a high furocumarins intake displayed a 5.67-fold risk (95%CI 3.74–8.61, *p* < 0.001) of developing erythema, followed by medium (OR 1.96 (1.52–2.53)) and low (OR 4.43 (2.93–6.71)) intakes, compared to no furocumarin intake. All the main foods rich in furocumarins consumed by our population achieved a statistically significant difference between omnivores, vegans and vegetarians (see Table 3).

## 4. Discussion

Several anecdotal cases had focused on vegetarian diet in humans with discordant results in establishing a potential cause-effect link between furocumarin-rich foods intake and a potential photosensitivity modification, conversely we found that a higher dietary intake of furocumarins in vegan and vegetarian diets is associated with greater skin sensitivity to NB-UVB phototherapy in psoriatic patients. Due to the increased prevalence of vegans and vegetarians among psoriatic patients [35], dermatologists started to evaluate potential differences in clinical outcomes and therapeutic management [36,37]. Although the Mediterranean diet, which is rich in vegetables, is regarded as beneficial for psoriatic patients [1], the impact of the single-food ingredients on phototherapy is entirely unknown. Furthermore, several foods included in the Mediterranean diet, such as celery, parsnip, carrot, parsley, citrus and figs, contain furocumarins, a natural, well-known photosensitizer able to trigger phytophotodermatitis in both humans and animals [20,38]. Since 100 g of parsnip or celery may contain up to 4–5 mg furocumarins, and a normal US diet only 1.3 mg furocumarins per day, a diet rich in some vegetables may contain up to 13 mg per day in the case of vegans and vegetarians, potentially sustaining photosensitivity [39,40].

Thus, our results support the notion that diet has a clinically meaningful impact on phototherapy management in psoriasis patients. In particular, clinicians treating psoriatic patients with phototherapy should include diet information in medical history. Vegans and vegetarians with psoriasis displayed higher photosensitivity than omnivores, and deserve *ad hoc* phototherapy management [28]. 

Phototoxicity has been widely proven in UVA wavelengths, since, in this UV range, psoralen DNA monoadducts are efficiently induced, but the greater the quantity ingested with a diet might convert the monoadducts to crosslinks [41,42]. Maximal phototoxic skin reactions in humans are induced by wavelengths between 334 and 425 nm, which perfectly falls into UVA spectrum (320–340 nm); however, in the presence of psoralens, such as furocumarins, phototoxicity may also appear in the spectrum of the less erythematogenic NB-UVB (311–313 nm). 

To further empower this statement, small amounts of dietary intake of furocumarins were reported to trigger photoxicity in both PUVA and NB-UVB [20,36]. 

We found an increased erythemal response to NB-UVB in subjects with high consumptions of furocumarins-rich foods; this is in contrast with Beattie et al., who found no effect when analyzing UVA-related photosensitivity [42]. 

Our results regarding erythemal response in psoriatic NB-UVB users undergoing different diet regimens and furocumarins intakes could be explained both quantitatively, since vegans and vegetarians consume more food containing furocumarins than omnivores, and qualitatively, since furocumarins bioavailability is affected by storage (fresh vs. frozen) and cooking (fresh vs. cooked) methods [41]. Interestingly, vegans and vegetarians consume foods containing high quantities of both antioxidants (astaxanthines and polyphenols) and furocumarins, but antioxidants may not counteract furocumarins-related photosensitivity/phototoxicity in our cohort of psoriatic patients. It is noteworthy that furocumarins have been shown to induce the secretion of melatonin in humans [43]; melatonin is a known antioxidant and anti-inflammatory hormone that exerts beneficial properties in the skin, and it is lower in psoriatic patients compared to healthy controls [44]. Although melatonin plasma levels were not measured in our cohort, this specific effect by furocumarins on melatonin, coupled with the effect on phototherapy response demonstrated here, may be of interest in the management of disorders, including psoriasis, which is itself associated with abnormalities in circadian rhythms [43].

This pilot study presents limitations, including a small sample size and a lack of skin metabolomic differences between the three considered groups. Furthermore, we used a solar simulator since the monochromator, the most precise instrument to measure MED on the market, was not present in the involved centers [45,46]. As current Italian guidelines did not suggest a specific instrument to measure MED [28], we used a solar simulator that, together with the monochromator, is internationally regarded as the gold standard [28]. 

## 5. Conclusions

In our study we found a different diet-related effect to NB-UVB, and this aspect acquired paramount importance, since vegans and vegetarians experience more erythema during NB-UVB, thus limiting the total number of phototherapy sessions in the considered period. Dermatologists should consider dietary assessment in patients’ clinical evaluation, and eventually also adapt phototherapy protocols to diet characteristics, since they seems to moderately influence patient management and clinical outcomes. Our clinical recommendations are summarized in Table 4. 

Further studies warranted to assess the impact of circadian rhythmicity on NB-UVB phototherapy sessions in clinical practice. 

## Figures and Tables

**Table 1:** Socio-demographic and clinical characteristics of omnivores, vegetarians and vegans.

Population Characteristics	Omnivores (*n* = 40)	Vegans (*n* = 38)	Vegetarians (*n* = 41)	*p*
Male, *n* (%)	19 (47.50)	18 (47.37)	16 (39.02)	0.68
Skin Phototypes				0.43
II	4 (10.00)	9 (23.68)	8 (19.51)	
III	17 (42.50)	16 (42.11)	19 (46.34)	
IV	19 (47.50)	12 (31.58)	14 (34.15)	
V	0 (0.00)	1 (2.63)	0 (0.0))	
Age (average (SD), years)	39.27 (9.24)	40.66 (8.28)	39.80 (8.96)	0.79
PASI (average (SD))				
T0	12.78 (2.83)	12.39 (2.95)	13.15 (3.27)	0.55
T1	1.95 (4.09)	8.87 (4.31)	4.59 (5.24)	<0.01
MED (average (SD), mJ/cm^2^)	33.62 (4.53)	21.18 (4.85)	28.90 (6.66)	<0.01
Treatments (average (SD)	15.07 (4.28)	12.13 (6.41)	14.61 (5.38)	0.40
Erythema (%)				<0.001
Absent	19 (47.50)	0 (0.00)	14 (34.15)	
Mild	17 (42.50)	10 (26.32)	14 (34.15)	
Moderate	0 (0.00)	12 (31.58)	6 (14.63)	
Severe	4 (10.00)	16 (42.11)	7 (17.07)	
PsA, *n* (%)	11 (27.50)	11 (28.95)	11 (26.83)	0.98
DAPSA (average (SD))	14.75 (2.99)	16.00 (2.05)	12.82 (2.32)	0.21

DAPSA: Disease Activity Index for PSoriatic Arthritis, MED: minimal erythematous dose, PASI: Psoriasis Area Severity Index, PsA: psoriatic arthritis, SD: standard deviation.

**Table 2:** Different intakes of the main photoactives between omnivores, vegetarians and vegans.

Level of Photoactives Ingested	Omnivores (*N* = 40)	Vegans (*N* = 38)	Vegetarians (*N* = 41)	*p*
Furocumarins, *N* (%)				<0.01
None	29 (72.50)	0 (0.00)	14 (34.15)	
Low	11 (27.50)	10 (26.32)	16 (39.02)	
Intermediate	0 (0.00)	7 (18.42)	7(17.07)	
High	0 (0.00)	21 (55.26)	4 (9.76)	
Carotenoids, *N* (%)				0.31
None	4 (10.00)	9 (23.68)	8 (19.51)	
Low	17 (42.50)	16 (42.11)	19 (46.34)	
Intermediate	19 (47.50)	12 (31.58)	14 (34.15)	
High	0 (0.00)	1 (2.63)	0 (0.0)	
Astaxanthines, *N* (%)				<0.01
None	15 (37.50)	0 (0.00)	0 (0.00)	
Low	16 (40.00)	13 (34.21)	17 (41.46)	
Intermediate	8 (20.00)	14 (36.84)	12 (29.27)	
High	1 (2.50)	11 (28.95)	12 (29.27)	
Polyphenols, *N* (%)				<0.01
None	17 (42.50)	0 (0.00)	0 (0.00)	
Low	18 (45.00)	19 (50.00)	16 (39.02)	
Intermediate	4 (10.00)	5 (13.16)	14 (34.15)	
High	1 (2.50)	14 (36.84)	11 (26.83)	

**Table 3:** Main foods rich in furocumarins consumed by the studied population.

Specific Foods Intake	Omnivores (*n* = 40), g/week	Vegans (*n* = 38), g/week	Vegetarians (*n* = 41), g/week	*p*
Parsley (average (SD)	1.0 (2.8)	601.3 (467.4)	35.3 (80.0)	<0.001
Grapefruit (average (SD)	7.5 (26.7)	1611.8 (1122.1)	113.9 (223.6)	<0.001
Lime (average (SD)	2.5 (11.0)	277.6 (245.3)	38.3 (60.9)	<0.001
Lemon (average (SD)	5.6 (17.4)	960.5 (495.0)	34.1 (74.5)	<0.001
Celeriac (average (SD)	0 (0)	717.1 (948.3)	67.1 (187.6)	<0.001
Parsnip (average (SD)	0 (0)	1063.2 (920.1)	23.2 (65.3)	<0.001
Celery (average (SD)	3.8 (13.3)	1355.3 (553.3)	169.5 (304.9)	<0.001
Orange (average (SD)	13.8 (40.8)	1726.3 (615.4)	402.2 (458.8)	<0.001
Cilantro (aver-age (SD)	0.3 (1.6)	96.2 (145.4)	6.7 (13.9)	<0.001
Carrots (average (SD)	8.2 (33.6)	1815.8 (711.1)	525.6 (785.4)	<0.001

**Table 4:** Clinical recommendations based on diet for NB-UVB phototherapy in patients with psoriasis.

NB-UVB CLINICAL RECOMMENDATIONS BASED ON DIET	
Omnivores	The starting dose is established after MED evaluation and corresponds to 70% of the MED; then, the dose is increased by 20% (if no erythema) or by 10% (in case of erythema) up to a maximum dosage of 3 J/cm^2^
Vegetarians and Vegans	The starting dose is established after MED evaluation and, in case of a low MED (20–25 mJ/cm^2^), corresponds to 40% of the MED; then, the dose is increased by 10% (if no erythema) up to a maximum single dose of 2.5 J/cm^2^. In case of erythema the dose is maintained constant

MED: minimal erythematous dose.

## Data Availability

Data available on request due to ethical restrictions. The data presented in this study are available on request from the corresponding author.
